# Heterogeneity and Function of Kupffer Cells in Liver Injury

**DOI:** 10.3389/fimmu.2022.940867

**Published:** 2022-06-27

**Authors:** Weiyang Li, Na Chang, Liying Li

**Affiliations:** Department of Cell Biology, Municipal Laboratory for Liver Protection and Regulation of Regeneration, Capital Medical University, Beijing, China

**Keywords:** macrophages, Kupffer cells, heterogeneity, function, liver injury

## Abstract

Kupffer cells (KCs) are key regulators of liver immunity composing the principal part of hepatic macrophages even body tissue macrophages. They reside in liver sinusoids towards portal vein. The micro-environment shapes KCs unique immunosuppressive features and functions. KCs express specific surface markers that distinguish from other liver macrophages. By engulfing gut-derived foreign products and apoptotic cells without triggering excessive inflammation, KCs maintain homeostasis of liver and body. Heterogeneity of KCs has been identified in different studies. In terms of the origin, adult KCs are derived from progenitors of both embryo and adult bone marrow. Embryo-derived KCs compose the majority of KCs in healthy and maintain by self-renewal. Bone marrow monocytes replenish massively when embryo-derived KC proliferation are impaired. The phenotype of KCs is also beyond the traditional dogma of M1-M2. Functionally, KCs play central roles in pathogenesis of acute and chronic liver injury. They contribute to each pathological stage of liver disease. By initiating inflammation, regulating fibrosis, cirrhosis and tumor cell proliferation, KCs contribute to the resolution of liver injury and restoration of tissue architecture. The underlying mechanism varied by damage factors and pathology. Understanding the characteristics and functions of KCs may provide opportunities for the therapy of liver injury. Herein, we attempt to afford insights on heterogeneity and functions of KCs in liver injury using the existing findings.

## Introduction

The liver acts as a crucial filtration system of body. It removes gut-derived products from the liver arterial and portal vein. To fulfil this, liver harbors the largest number of tissue macrophages, named Kupffer cells (KCs). KCs are first reported as components of vascular endothelial cells by Karl Wilhelm von Kupffer in 1876 (1). In 1974, Wisse distinguishes KCs from endothelial cells and defines them as liver sinusoids residing macrophages (2). Then, KCs are identified as F4/80^+^ (mouse) or CD68^+^ (human) macrophages that emerged in liver and maintained their number mainly by proliferation (3). In recent years, people have recognized that liver macrophages are composed by not only KCs, but also other macrophages (such as bone marrow monocyte-derived macrophages (BMMs) or capsular macrophages). Meanwhile, KC definition is updated. In the new definition, mouse KCs are liver macrophages that specifically expressing CLEC4F regardless of origins (4). Besides, CLEC2 (5), TIM4 (6) and VSIG4 (7) are also recognized as mouse KC markers. Among them, VSIG4 has recently been reported to be conservative among mouse and human (7). Accordingly, KCs can be distinguished from other macrophages (8).

In recent years, researchers pay more attention to KC features and functions, and have got many impressive results on this issue. The heterogeneity of KCs has been unveiled, especially in mouse. In this review, we summarize and present insights on heterogeneity and function of KCs (liver macrophages that express KC markers) under new definition.

## KC in Homeostasis

### Heterogeneity of KC Origin

**Figure 1:**
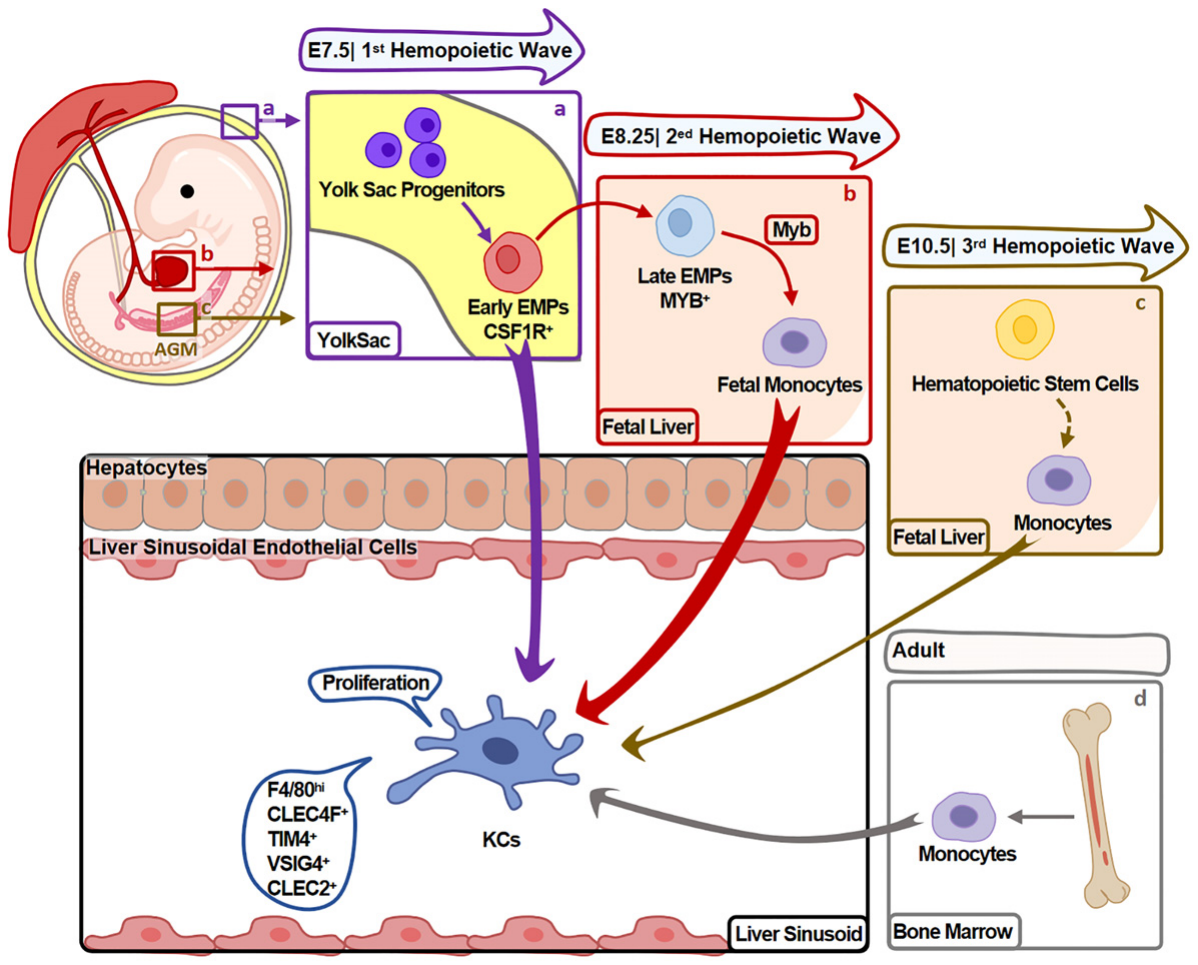
Heterogeneity of KC Origin. KCs mainly develop during hemopoiesis. **(A)** In the first hemopoietic wave (around E7.5), YS-macrophages are differentiated from EMPs in YS without experiencing monocyte stage. Partial YS-macrophages then migrate and reside in fetal liver, further specialize into KCs. **(B)** In the second hemopoietic wave (around E8.25), late EMPs differentiate into KCs undergo a monocytic intermediate in fetal liver. **(C)** In the third hemopoietic wave (around E10.5), hematopoietic stem cells originate from the aorta-gonads-mesonephros (AGM) region and migrate to fetal liver. They give rise to monocytes that differentiate into KCs during perinatal period. KCs developed in this process contribute minor to adult KCs. **(D)** Fourthly, in adults, BM monocytes also give rise to minor KCs. Arrow width represents the contribution of relative progenitors to KCs.

KCs mainly develop in the three hemopoiesis waves (**Figure 1**) and the developmental program is conserved between mouse and human (9). In mouse, the first wave (primitive hemopoiesis) starts around embryonic days 7.5 (E7.5). Early erythro-myeloid progenitors (EMPs)(CSF1R^+^) originated from yolk sac (YS) give rise to YS-macrophages. Then, partial YS-macrophages migrate into fetal liver and develop into KCs (10). In the second wave (transient hemopoiesis), late EMPs (MYB^+^) seed in fetal liver and give rise to fetal monocytes, which specialize into KCs before E16.5 (11). In the third wave (definitive hemopoiesis), hematopoietic stem cells arise intra-embryonically around E10.5 and migrate into fetal liver. Then, hematopoietic stem cells give rise to KCs undergo a monocytic intermediate during perinatal period (11).

Fate-mapping studies suggest that the first two waves arise majority of KCs. Hematopoietic stem cells-derived KCs (in the third wave) contribute to a minor part of KCs, and do not replace KCs from the first two waves. These embryo-derived KCs (Em-KCs) inhabit in liver for the whole life and maintain by proliferation (11, 12).

In adults, bone marrow monocytes also contribute to maintain KC pool when Em-KCs are insufficient to maintain it (13). Studies have demonstrated that when Em-KCs are depleted by clodronate liposomes (CLs) (14, 15), diphtheria toxin (4), anti-TIM4 antibody (6) or high dose irradiation (16), monocytes are recruited and differentiate into KCs to replenish the vacancy. In patients received liver transplant, recipient macrophages are also emerged in transplanted livers (17). Therefore, monocytes might differentiate into KCs in both mouse and human. But it should be noticed that these bone marrow monocyte-derived KCs (BM-KCs) are different from BMMs in injured liver. BM-KCs express KC markers (including CLEC4F, CLEC2, VSIG4, TIM4), while BMMs mainly highly express CCR2 and show no KC marker expressions (6, 18).

Besides, KC pool remodeling schedule has been reported in two different mouse models: (a) TIM4 antibody-depleted KC mouse model. In this model, KCs are fully depleted in 2 hours followed by the replenishment of immature BM-KCs (TIM4^-^F4/80^+^). TIM4^-^BM-KCs are matured as TIM4^+^BM-KCs after 30 days (6). (b) *Clec4f*-DTR transgenic mouse model. Using this model, two groups provide information from distinct aspects. Martin Guilliams and colleagues report that dead Em-KCs initiate the process by activating liver sinusoidal endothelial cells (LSECs) and hepatic stellate cells (HSCs) around. Then, activated LSECs and HSCs recruit Ly6C^+^monocytes from circulation at day 1 post Em-KCs depletion. Monocytes then acquire KC phenotype and loss Ly6C expressions gradually in 6 days (18). Christopher Glass group describe more details in transcriptional level. They report that KC lineage determining transcription factors (LDTFs) (NR1H3, SPIC, ID3) are increased while monocyte gene (CCR2) is decreased in replenished monocytes within 12 hours post depletion. In the following 36 hours, more KC LDTFs (MAF, TFE, MAFB, TFEC, etc) gradually express accompanied by the decrease of monocyte related transcription factors (C/EBP, RUNX, SP2, etc.). At day 3 to 7, KC markers CLEC4F and TIM4 are up-regulated (19).

Three signals from liver sinusoids are reported to mediate BM-KCs differentiation. Firstly, DLL4/Notch signaling initiates monocytes differentiation by inducing the increase of KC LDTFs (18). Secondly, TGFB1/BMP9-SMAD4 signaling maintains KC phenotype. Thirdly, KC lineage transcription factor LXRa (encoded by *Nr1h3*) are also crucial for KC phenotypic maintenance (19). Moreover, two recent studies identified that BMP9/10-ALK1-SMAD4 signaling also regulate KC identity, proliferation and functions (7, 20). But the contribution of TGFB1 in KC identity is controversial: Christopher K. Glass group suggest that TGFB1-SMAD4 signaling maintains KC phenotype in the presence of DLL4 (19). However, Tang group deny the contribution of TGFβ1 using *Tgfbr2*^fl/fl^*Clec4f*^Cre^ mouse (20). Whether other receptors in TGFβ superfamily (e.g. BMP receptors) (20) mediate TGFβ1 function is less studied. Therefore, the significance of TGFB1 signaling in KC identification need more in-depth studies.

Collectively, the origins of KCs are complex. They are mainly originated from three hemopoiesis waves. But BM monocytes also contribute to KC pool when Em-KCs are insufficient to maintain it. The ability of BM monocytes differentiating into KCs has been validated. But the process of KC replenishment is varied and depends on KC depletion methods. The mechanism underlying monocyte-KCs differentiation still needs further study.

### Heterogeneity of KC Phenotype and Function

Although the common functions of KCs have been reported as removing bacteria and apoptotic cells (21–24), antigen-presenting (25), and iron/lipid metabolism (4, 26). The consensus is that KCs are heterogeneity in phenotype and function. But no criteria are accepted for KC classification till now. We here summarize the existing findings on mouse KCs (**Table 1**) and human KCs will be described in part 2 (Human KCs).

**Table 1:** KC heterogeneity and function in homeostasis.

Phenotype	Marker	Feature	Function	Reference
M1 KCs (Classically Activated)	CD86, iNOS, CD80	(a) Highly expressing: IL1β, TNFa, IL6, IL12p70, CCL2, CCL4, CCL3, CCL11, CXCL1, CXCL2, CXCL3; (b) Transcription Factor: STAT1	(a) Pro-inflammatory; (b) Antigen presentation; (c) Th1 immune reaction; (d) Pathogen elimination	(27, 28)
M2 KCs (Alternative Activated)	ARG1, MRC1, MGL1, CD163	(a) Highly expressing: TGFB1, IL10, CCL17, CCL22; (b) Transcription Factor: STAT6, STAT3	(a) Pro-resolution; (b) Anti-inflammation; (c) Th2 immune response; (d) Phagocytosis;	
Em-KCs	CLEC4F, VSIG4, CLEC2, TIM4, CD5L	(a) Proliferation; (b) Compose the majority of healthy KC pool; (c) Dead upon liver injury	(a) Phagocytosis; (b) Removing apoptotic cells, senescent erythrocyte, red blood cells, pathogens, immune complexes; (c) Lipid/iron metabolism; (d) Immunosuppression; (e) Antigen presentation; (f) Responding to LPS and *Leishmania* infection	(21–26)
BM-KCs	CLEC4F, VSIG4, CLEC2, TIM4, CD5L	(a) Proliferation; (b) Contributing to minor of healthy KC pool; (c) In irradiation-exhausted KC mouse model: Lacking 42 genes of Em-KCs; (d) In CLs-depleted KC mouse model: Need 30 days to fully obtain KC genes	(a) Phagocytosis; (b) Low lipid storage*; (c) Pro-inflammation*; (d) Stronger phagocytosis of *N. meningitidis* and *L. monocytogenes*; (e) Clearing red blood cells; (f) Responding to LPS and *Leishmania* infection	(4–6, 14, 16)
Radioresistant KCs	*Cdkn1a*^hi^	(a) Radioresistance; (b) Embryo-derived	Radioresistance in lethal irradiation	(15)
KC1	CD206^lo^ ESAM^-^	Occupy ~80% of Em-KCs	(a) Phagocytosis; (b) Immune regulating	(29)
KC2	CD206^hi^ ESAM^+^	(a) Occupy ~20% of Em-KCs; (b) Highly express EC genes	(a) Phagocytosis; (b) Regulating Metabolism	(29, 30)
Human KCs	CD163^+^MARCO^+^CD5L^+^TIM4^+^		(a) Anti-inflammation; (b) Anti-tumor; (c) Regulating immune response	(31–33)
Human KCs	CD32^int^CD68^+^CD14^+^		Regulating immune response	(34)
Human Em-KCs	CD49a^+^ CD68^+^ VSIG4^+^ MARCO^+^	Highly expressing TNF, IL12 and IL10 and cannot be up-regulated by LPS	–	(35)
Human BM-KCs	CD49a^-^ CD68^+^ VSIG4^+^ MARCO^+^	TNF, IL12 and IL10 expression are increased by LPS stimuli	–	

*TIM4^-^BM-KCs functions; -, Need to study.

Historically, KCs are classified using M1 (classical activated macrophages, pro-inflammation) and M2 (alternative activated macrophages, pro-repairing) dogma *via* their phenotype under different stimuli. For example, KCs are activated into M1 phenotype by lipopolysaccharide (LPS), IFN-gamma or M-CSF, characterizing by highly expressing pro-inflammatory genes IL1B, TNFα and iNOS (36). While IL4 or GM-CSF may polarize KCs into M2 type that highly express ARG1, IL10 and MRC1 (27, 28). However, later studies found that M1-M2 dichotomy is insufficient to classify KCs, especially in liver injury (37, 38). Therefore, more studies attempt to seek an accurate method to classify KCs.

Some studies use the origin of KCs to describe their heterogeneity. As described in part 1.1, KCs are composed by BM- and Em-KCs. Based on the existing studies, BM- and Em-KCs seem to be similar but not identical. First, BM- and Em-KC gene profiles are inconsistent. In irradiation-exhausted KC model, BM-KCs lack in 42 Em-KC genes related to iron homeostasis (16). While iron/lipid metabolism related genes are at similar levels in BM- and Em-KCs in *Clec4f*-DTR transgenic mice (4). Second, functions of BM- and Em-KCs are different. As described above, BM-KCs might be shortage in iron homeostasis for the lack of related genes compared with Em-KCs in irradiation-exhausted KC mouse model (16). In the same model, BM- and Em-KCs show different phagocytic ability based on ligand specificity exist. BM-KCs exhibit stronger ability to engulf *N. meningitidis* and *L. monocytogenes* compared to Em-KCs, while the phagocytic capacities for red blood cells and *S. typhimurium* clearing are similar (16). The findings in CLs-depleted KC model might explain the difference on phagocytic ability. It reports that BM-KCs need to undergo a 30 to 60 days’ “education” process to obtain similar phagocytosis capacity to Em-KCs (14). However, whether the differences between studies are caused by mouse model or experimental conditions need further studies.

Besides, Em-KCs are also considered to be heterogeneity. In irradiation-exhausted model, a cluster of radioresistant KCs (*Cdkn1a*^hi^) are identified (15). However, specific functions of the radioresistant Em-KCs need in-depth studies. Besides, two distinct Em-KC clusters have been unveiled using scRNA-seq: KC1 (CD206^lo^ESAM^–^, occupy ~80% of Em-KCs) and KC2 (CD206^hi^ESAM^+^). KC1 shows stronger immune signature while KC2 is bias to regulate metabolism. Meanwhile, KC1 and KC2 show similar phagocytic capacities and distributions in liver (29). In recent research, KC2 is considered as doublet of KCs and LSECs *via* CITE-Seq analysis (7). Herein, further studies still need to explore and describe Em-KCs heterogeneity.

Finally, KC heterogeneity might also be determined by localization. In terms to the anatomical structure and transcriptional differences, hepatic lobule is heterogeneity and is separated into periportal, mid and pericentral zones (39). Based on it, the metabolic and immune zonation of hepatic lobule have been studied (40, 41). Therefore, studies are also trying to unveil KC heterogeneity based on their localization. The existing findings support the view that KCs prefer to locate in the periportal and mid zones (adhere to the portal vein) (7, 39, 40). The portal vein-adhering localization of KCs is maintained by endothelial MYD88-mediated CCL9 gradients (40) and gut microbiota derived commensal D-lactate (42).

## Human KC

Similar to mouse KCs, human KCs also locate around portal vein (31) and are considered to be heterogeneity. But the research progression has been at a slow pace by the limitations of technology. Herein, we conclude the available findings for human KCs (**Table 1**).

First, the common feature and function of human KCs have been studied. But it has not reached agreement since the lack of unified markers to identify human KCs. In three separate scRNA-seq studies, human KCs are identified as CD163^+^MARCO^+^CD5L^+^TIMD4^+^ (31–33). Functionally, these KCs are potential to anti-inflammation, anti-tumor and regulating immune (31–33). Comparing to MARCO^-^ macrophages, KCs express less inflammatory (TNFα) but more immunosuppressive genes (e.g. PD-L1) under LPS or IFN-gamma stimuli (31). Another study suggests that KCs are characterized as CD32^int^CD68^+^CD14^+^ and have potential to regulate immune response (34). In recent study, VSIG4 is considered as the best maker of human KCs (7). Therefore, human KCs remain need accurate and canonical definition.

Second, human KC heterogeneity is also reported. In accordance with mouse KCs, human KCs are also separated into Em- and BM-KCs. CD49a has been suggested to distinguish them, while Em-KCs are CD49a^+^ (35). Em-KCs express high levels of pro-inflammatory TNFα, IL12 and anti-inflammatory IL10, suggesting their dual role in inflammation. But LPS cannot affect these cytokines expressions in Em-KCs. In contrast, the three cytokines are at low levels in BM-KCs while can be up-regulated by LPS (35). Therefore, Em-KCs seems to be the functional cluster in homeostasis while BM-KCs function in injury.

Collectively, the following problems are exposed in current research: Firstly, human KC canonical markers are still lacking. Since human KCs are difficult to distinguish from other macrophages, their functions are also difficult to study. Secondly, human KC heterogeneity (especially origins) is hard to study due to technical limitations. Thirdly, knowledge of mouse KCs cannot be transferred directly to human KCs because of species difference. Fourthly, the individual difference of human beings poses the difficulty to study the universality feature and function of human KCs, especially under diseases.

## KC in Acute Liver Injury

### Acetaminophen (APAP)-Induced Acute Liver Injury

APAP is a widely used analgesic-antipyretic drug, but is also a major cause of acute liver injury. APAP overdose leads to hepatocytes apoptosis which triggers activation of immune cells including KCs (43, 44).

KC number changes dynamically in APAP liver injury. In APAP-stimulated liver, KC number reduces gradually within 48 hours. After 72 hours, residual KCs start to recover by self-renewal (45).

**Figure 2:**
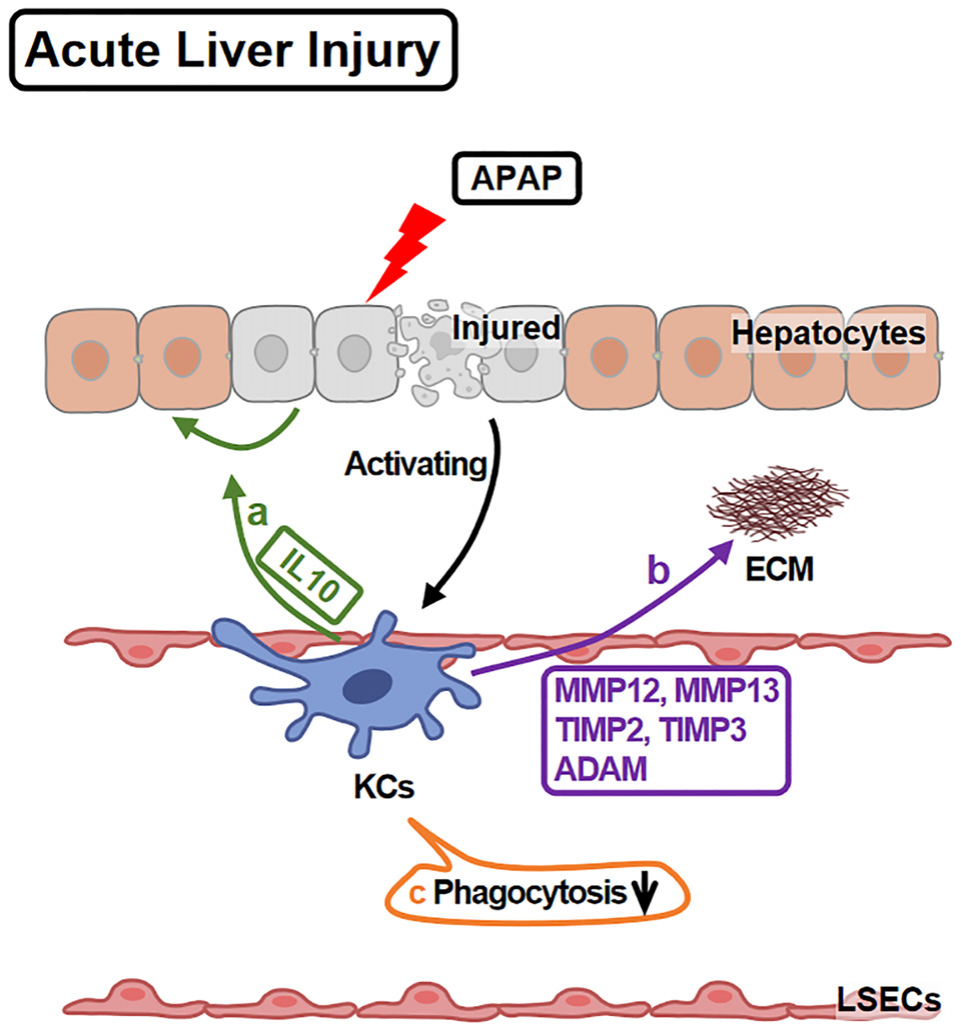
KCs in APAP-induced Acute Liver Injury. APAP overdose leads to hepatocyte injury and death which activate KCs. Activated KCs initiate the repairing response: **(A)** KCs secrete IL10 to promote injured hepatocyte regeneration and survival. **(B)** KCs release MMP12, MMP13, TIMP2, TIMP3 and ADAM, remodeling ECM. **(C)** However, KCs phagocytotic ability is injured during these processes.

Functionally, KCs play protective and pro-repairing roles in APAP-liver injury, since KCs depletion leads to more serious injury and slower recovery (14) (**Figure 2**). The underlying mechanisms include: First, KCs promote hepatocytes survival *via* IL10 (46). Second, KCs facilitate tissue repair through regulating extracellular matrix (ECM) remodeling. KCs express ECM remodeling-related genes, including MMP12, MMP13, TIMP2, TIMP3 and ADAM23 (47), among which, MMP12 is well studied. MMP12 helps to restore hepatocyte proliferation and to reduce necrosis (48). KCs increase MMP12 expression (45) to degrade elastin (a major component of ECM) and facilitate liver repairing (49).

### Infected Liver Injury

As major component of liver immunity, KCs play important role in infected liver injury. KCs uptake and eradicate pathogens in this process, which usually causes KC death. In hepatitis B virus (HBV) induced acute liver injury, KCs are reduced in number while promote anti-virus response (50). Besides, scRNA-seq analysis show that CD206^+^ESAM^+^ Em-KCs respond to IL2 and cross-present antigens to enhance T cell mediated HBV killing (30). In vaccinia virus and murine cytomegalovirus infection, KCs are reduced and replenished by both proliferation and infiltrated monocytes. Type I IFN response is suggested to modulate monocytes-KCs differentiation (51). In adenovirus infection, KCs bind and uptake adenovirus *via* VSIG4 and complement C3 (52).

In *Listeria monocytogenes* infection, KCs engulf bacteria and cause their own necroptosis. KC necroptosis recruits monocytes, and induces aggregation of Th2 cytokines including basophil-IL4 and hepatocyte-IL33. Monocytes differentiate into KCs and obtain proliferative property under IL33/IL4 stimulation. Functionally, BM-KCs attenuate inflammation and restore liver homeostasis (53).

### Liver Ischemia-Reperfusion Injury (LIRI)

LIRI refers to further aggravation of ischemic injury after blood perfusion recovery, causing by multiple risks including liver transplantation. LIRI induces the necrotic depletion of KCs (54). KC number reduces in 6 hours after LIRI while recovers at day 3 and increases at day 7. The recovered and increased KCs are immature BM-KCs featured by TIM4-lacking (6).

During LIRI, KCs mediate inflammation by IL1B, which is induced by inflammasomes NLRP3 and AIM2 (55, 56). In parallel, a CSF3^+^KC cluster in rat shows potential to promote inflammation by multiple cytokines and chemokines under IR challenge (57). On the other hand, KCs also regulate LIRI recovery. KCs resolve liver inflammation by TIM4-mediated IL10 up-regulation and TNFα down-regulation. Additionally, KCs also promote the recovery *via* TIM4-mediated efferocytosis (6).

### Acute Liver Injury Induced by Other Risks

In acute carbon tetrachloride (CCl_4_)-liver injury, KCs are impaired resulting in the decrease of IL-6, further delayed liver regeneration (58). In LPS induced acute liver injury, KCs induce liver inflammation and hepatocyte death by up-regulating TNFα (59), although the up-regulations of both pro- and anti-inflammatory transcription factors are detected (60). Meanwhile, LPS-activated KCs increase the mortality because of the low level of CETP (61).

Collectively, KCs play a complicated dual role in acute liver injury, which might be depended on damage factors. But there are two common points: First, KC number is reduced under acute injury, which leads to monocyte infiltration. Second, KC functions are impaired in different damage. Therefore, replenishing KC number and functions might be a therapeutic strategy for acute liver injury.

## KC in Chronic Liver Injury

### Non-Alcoholic Steatohepatitis (NASH)

NASH is a severe liver disease that can advance to fibrosis, cirrhosis even hepatocellular carcinoma. The characters of NASH include metabolic disorders, liver inflammation and steatosis.

In NASH liver, Em-KCs are reduced with the increase of diet cholesterol content (62–64). It might be caused by NASH-induced cell apoptosis or death (5, 65). Following KC reduction, monocytes are recruited and differentiate into BM-KCs (5, 64). At the same time, residual Em-KCs expand by proliferation (63) while CD206^hi^ESAM^+^ Em-KCs (KC2 mentioned in Part 1.2) maintains stable in number (29).

**Figure 3:**
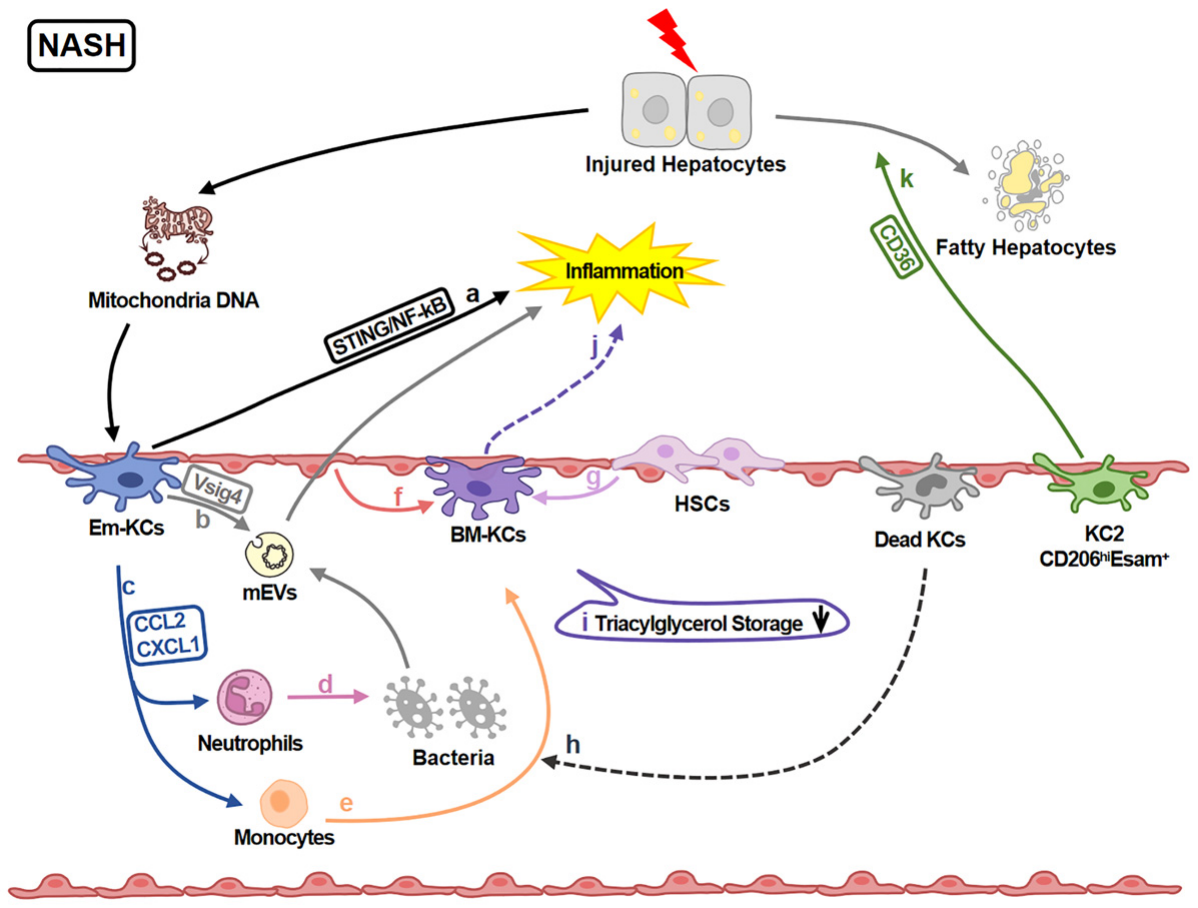
KCs in NASH-related Liver Injury. In NASH livers, KCs regulate inflammation and lipid metabolism. **(A)** KCs-STING/NF-kB signaling is activated by mitochondria DNA from injured hepatocytes and amplify the inflammation. **(B)** KCs inversely engulf gut mEV (microbial DNA containing extracellular vesicles) *via* VSIG4 and attenuate inflammation. **(C)** KCs also secret chemokines CCL2 and CXCL1 recruiting neutrophils and monocytes. **(D)** Neutrophils help killing pathogens like bacteria. **(E)** Monocytes can differentiate into BM-KCs. **(F, G)** The differentiation process is regulated by LSECs and HSCs. **(H)** Dead KCs are the initiators of the replenishment by opening KC niche. **(I)** The triacylglycerol storage capacity of BM-KCs is decreased comparing to Em-KCs. **(J)** Meanwhile, BM-KCs promote inflammatory response. **(K)** KC2 (CD206^hi^ESAM^+^KCs) regulate lipid metabolism *via* CD36 leading to the damage of hepatocytes.

KCs play various roles in regulating liver injury in NASH (**Figure 3**). 891 genes in KCs are reported to be up-regulated under NASH. These genes are associated with functions of ECM remodeling, lipid metabolism, bacterial clearance and recruitment of circulating monocytes (62). Moreover, KCs amplify or attenuate liver inflammation. Mitochondrial DNA from apoptotic hepatocytes activates STING/NF-kB signaling pathway in KCs and leads to inflammation amplification (66). KCs also recruit monocytes and neutrophils *via* CCL2 and CXCL1. Monocytes aggravate inflammation while neutrophiles contribute to killing bacteria (67). KCs attenuate inflammation by removing gut microbial products *via* VSIG4 (68, 69).

Additionally, functions of different KC sub-clusters are also reported. For example, KC2 aggravate liver injury *via* CD36 (29); Em-KCs are more efficient for triglyceride storage (5, 70) while BM-KCs are more inflammatory (5). It still needs further study to explore whether these differences are caused by origins, KCs impairment and/or the different state of KCs (such as immature or mature).

Therefore, KCs play multiple roles in NASH. The details of KC functions, especially based on their heterogeneity, still need deeply study.

### Cholestatic Liver Injury

Cholestatic liver injury involves a variety of disorders of bile flow and/or formation. KCs are reduced in primary biliary cholangitis (a chronic cholestatic liver disease) (71). In bile duct ligation (BDL) mouse model, KC depletion results in a reduction of hepatocyte regeneration and more serious injury (72, 73). However, liver fibrosis is alleviated by KC deletion (73). In-depth study suggests that IL6 might be a potential functional molecule (72). On the other hand, BDL impairs the clearance function of KCs, resulting the reduction of gut-derived LPS clearance and the aggravation of inflammatory damage (74, 75). In parallel, KC scavengers MARCO and CD5L are reduced in human biliary atresia (76). Decreased clearance function might be a reason of the susceptibility to bacterial infection after BDL injury (74).

### Toxic Liver Injury

As the major organ for detoxification, liver is easily to be injured by toxicant (such as chemicals, drugs and alcohol). In this part, we mainly focus on chronic toxic liver injury since acute toxic liver injury has been discussed in Part 3.

In CCl_4_-chronic liver injury model, KC number is reduced and is partially replenished by Ly6C^lo^ monocytes during repairing (77). Functionally, KCs amplify inflammation by recruiting pro-inflammatory macrophages *via* TREM1 in early stage of injury (78). KCs phagocytic and pathogen killing potentials might be decreased for complement C6, MARCO and TIM4 in KCs are down-regulated (79). But in another study, KC-TIM4 is increased (80). Herein, more studies are needed to demonstrate how KC phagocytic capability changes during toxic liver injury.

In alcohol injured livers, alcohol also induces KC number decreasing (81). KCs are activated by apoptotic hepatocyte-derived mitochondrial DNA *via* TLR3. Activated KCs produce high level of IL1B which induces IL17A releasing from T cells (82) and promotes hepatocellular carcinoma development (83).

### Chronic HBV Infection

In part 3.2, we have discussed the importance of KCs in acute HBV infection. In chronic HBV infection, KCs also play protective role in various ways. Firstly, KCs produce IL1B *via* activated NLRP3 (84) to attenuate the susceptibility of HBV infection (85). Secondly, KCs recognize virus and enhance natural killer cells activation *via* IL18 (86). Thirdly, KCs act as antigen-presenting cells to activate T cells (87). Additionally, KCs contribute to liver immune tolerance by attenuating T cell proliferation (88), attenuating B cell antibody production (89), suppressing CD8^+^T cell cytotoxicity and promoting Treg expansion (90). Inversely, HBV also employs different strategies to modulate KCs to favour the establishment of infection, such as impairing IL-1B production (84) and promoting IL-10 releasing (91).

## Liver Fibrosis and Cirrhosis/Portal Hypertension

Liver fibrosis, a common pathological process of chronic liver injury, is characterized by the excessive deposition of ECM (especially collagen). Liver fibrosis further progresses to cirrhosis if it has not been well treated. Portal hypertension is a major cause of morbidity and mortality in cirrhosis (92). In human cirrhosis, KC number has been reported to be unchanged (33, 93) while rat KCs are reduced upon cirrhosis (94). A recent study reports that infiltrated monocytes differentiate into BM-KCs upon liver stromal cells-IL6 stimuli (95).

**Figure 4:**
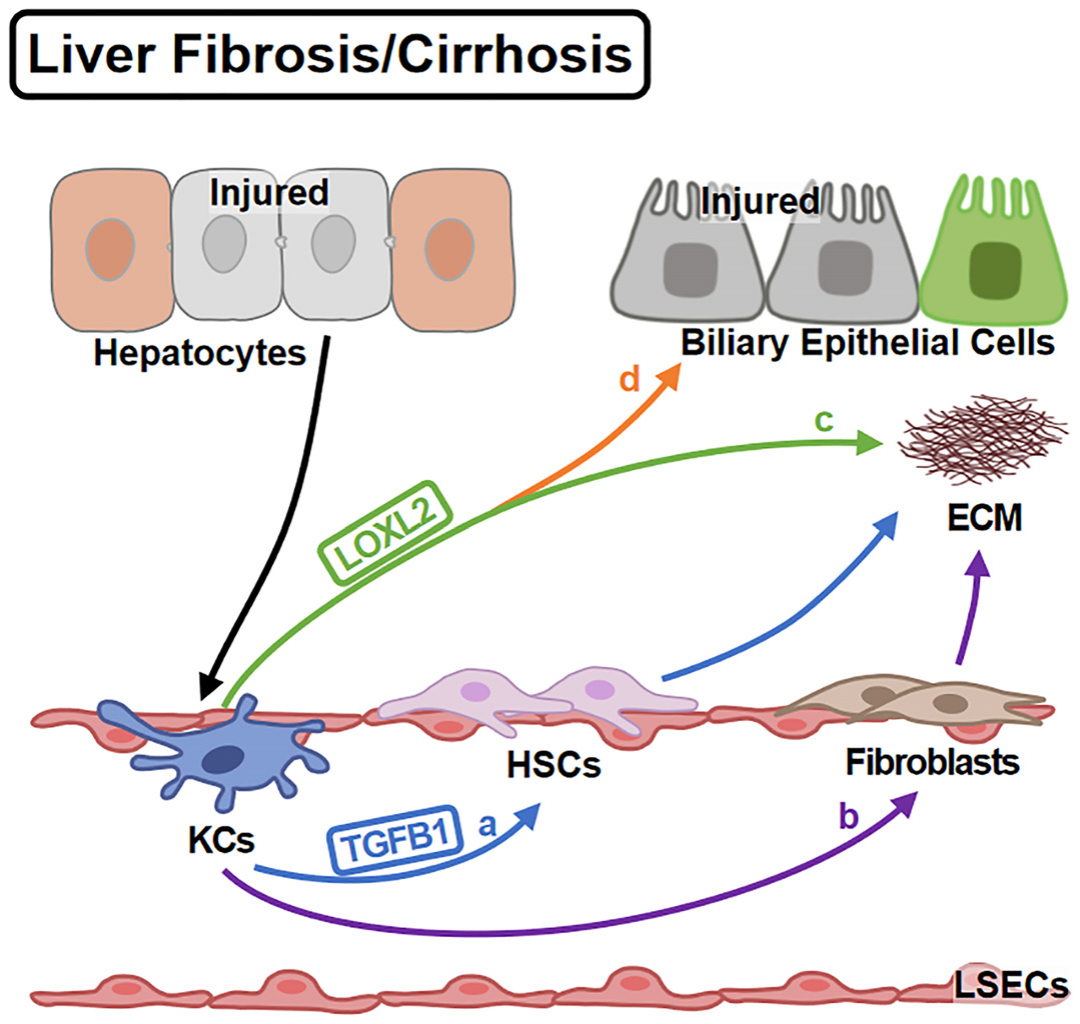
KCs in Liver Fibrosis/Cirrhosis. Liver fibrosis is the over-repair in chronic liver injury. KCs are activated by injured hepatocytes and promote fibrosis in different ways: **(A)** KCs promote HSC activation *via* TGFB1. **(B)** KCs themselves can differentiate into fibroblasts contributing to ECM deposition. On the other hand, **(C)** KCs mediate the cross-linking of collagen *via* LOXL2. **(D)** KCs also induce the impairment of biliary epithelial cells by LOXL2.

KCs regulate ECM deposition in various ways (**Figure 4**). Firstly, KCs affect collagen production by regulating HSC (the major cellular source of collagen) activities. KCs promote HSC activation and collagen production *via* TGFB1 (80). For another, KCs contribute to HSC survival by EGFR and TNFR1A/1B signaling (79). Secondly, KCs regulate ECM remodeling directly. KCs promote collagen cross-linking and scar formation (96) by LOXL2 (97). Inversely, KCs produce MMP9 to promote collagen resolution (98). Consistently, KC infusion shows anti-fibrotic effects (99).Thirdly, KCs also transdifferentiate into fibroblast-like cells contributing to ECM deposition (100).

In human cirrhosis, phagocytic and anti-bacteria abilities of KCs are weakened (93) which might cause the susceptibility to bacterial infection and the cirrhotic death (101). At the same time, activated KCs might help increase portal venous pressure for the level of soluble CD163 (the sensitive marker of KC activation) is associated with portal venous pressure gradient (102). Activated KCs also increase portal pressure by inducing vasoconstrictor including cysteinyl leukotriene (103). On the other hand, human KCs might not contribute to fibrogenesis directly for they are away from fibrotic areas (33). The contradiction in human KCs studies might be caused by the influence of other liver macrophages, because of the lacking of canonical markers to distinguish them, especially in early studies.

## Hepatocellular Carcinoma (HCC)

HCC is an inflammation associated cancer caused by multiple etiological factors. KCs are reduced in mouse (104) and human (105) HCC. Human KC reduction is caused by tumor cell-CCL2 (105). However, mouse KCs could be replenished for CLEC4F expression is unchanged (106). Herein, the change of KCs pool in HCC still needs further study.

Functionally, KCs promote neutrophil-mediated liver toxicity through IL12 (107). KCs also attenuate T cell responses thereby promote tumor growth and decrease anti-PD-1 therapeutic sensitivity (108). While up-regulating microRNA-206 expression might attenuate the tumor-promoting effect on of KCs (109). Besides, CD163^+^CD206^+^FOLR2^+^KCs co-localize and interact with PLVAP^+^ endothelial cells and immunosuppressive T cells, further maintained the immunosuppressive micro-environment (110). On the other hand, cancer cells might regulate KC activities directly since liver macrophage activity and function are affected by cancer cells (111). Cancer cells also change macrophage cytokines-releasing (112) and metabolism (113) which further promote HCC progression. Cancer cell-KCs crosstalk exacerbates HCC by initiating a vicious circle which reinforces each other.

## Conclusion and Further Prospect

As one of the important components of liver, KCs perform important role in health, acute/chronic liver injury, liver fibrosis/cirrhosis and HCC. In healthy liver, KCs help to maintain liver homeostasis. In injured liver or HCC, KCs are usually reduced, but the detail and reason for reducing need in-depth study. KC functions in injured liver are complicated (**Table 2**). Further studies are needed to confirm the universal between different injuries. In recent years, the improvements of technology (such as scRNA-seq) provides more information and details about KC heterogeneity in health and diseases. But the conclusions are controversial and lack an accepted clustering criterion. In conclusion, it is necessary to further clarify KC functions (especially based on their heterogeneity) in liver diseases. These studies will be helpful in understanding the mechanism of liver diseases and developing new therapeutic target.

**Table 2:** KC heterogeneity and function in Liver injury.

Injured Risk		KC Number	Replenished by BM or self-renewal?	Functional KC Phenotype	KC Function	Reference
Acute	APAP	↓ in 48 hours; Recover after 72 hours	Self-renewal	All	(a) Engulfing and clearing apoptotic cells; (b) Promoting hepatocyte regeneration; (c) Regulating ECM remodeling	(14, 45, 47, 104)
	HBV	↓	–	CD206^+^ESAM^+^	Enhancing T cell mediated HBV killing	(30)
	Vaccinia virus and murine cytomegalovirus	↓	Both	All	–	(51)
	Adenovirus	↓	–	All	Uptaking adenovirus	(52)
	*Listeria monocytogenes*	↓	BM	BM-KCs	(a) Attenuating liver inflammation; (b) Promoting liver homeostasis restoration	(53)
	LIRI	↓ in 6 hours; Recover at day 3; ↑ at day7	BM	BM-KCs (TIM4^-^)	(a) Pro-inflammation; (b) Attenuating LIRI resolution	(6)
				Em-KCs and BM-KCs	(a) Pro-inflammation; (b) Promoting inflammation resolution; (c) Efferocytosis	(6, 55–57)
	CCl_4_	–	–	All	Promoting liver regeneration	(114)
	LPS	–	–	All	Inducing inflammation and hepatocyte death;	(59–61)
Chronic	NASH	↓ or Unchanged	Both	Em-KCs	(a) Triglyceride storage; (b) Pro-inflammation;	(5, 66, 67)
				BM-KCs	Pro-inflammation;	(5)
				CD206^hi^ESAM^+^	Aggravating liver injury	(29)
				All	Engulfing pathogens	(68, 69)
	BDL	↓	–	All	Promoting hepatocyte regeneration	(72, 73)
				Impaired KCs	Relating to the bacterial infection	(74, 75)
	Primary Biliary Cholangitis	↓	–	–	–	(71)
	CCl_4_	↓	Both	All	(a) Pro-inflammation; (b) Pro-fibrosis; (c) Promoting HSC activation and survival; (d) Attenuating fibrosis	(79, 80)
	Alcohol	↓	–	All	Promoting the injury progressing to hepatocellular carcinoma	(81, 82)
	HBV	–	–	All	(a) Attenuating the susceptibility of HBV infection; (b) Regulating immune response	(84–91)
Human Biliary Atresia		–	–	Impaired KCs	MARCO and CD5L expression are down-regulated	(76)
Human Cirrhosis		Unchanged	–	MARCO^+^CD163^+^	Up-regulating portal venous pressure	(93, 101–103)
HCC				CD163^+^D206^+^ FOLR2^+^	Maintaining the immunosuppressive micro-environment	(110)
				All	(a) Promoting neutrophils mediated liver toxicity; (b) Attenuating T cell responses; (c) Crosstalking with cancer cells	(107, 108, 111, 113)

-, Need to study. ↑，Increase； ↓, Reduction.

## Author Contributions

WL and NC drafted the manuscript and designed the figures. LL and NC made the final corrections. LL applied the funds. All authors contributed to the article and approved the submitted version.

## Funding

This work was supported by grants from the National Natural and Science Foundation of China (81970532, 82170622).

## Conflict of Interest

The authors declare that the research was conducted in the absence of any commercial or financial relationships that could be construed as a potential conflict of interest.

## Publisher’s Note

All claims expressed in this article are solely those of the authors and do not necessarily represent those of their affiliated organizations, or those of the publisher, the editors and the reviewers. Any product that may be evaluated in this article, or claim that may be made by its manufacturer, is not guaranteed or endorsed by the publisher.

## Abbreviations

APAP, Acetaminophen; BDL, bile duct ligation; BM-KCs, bone marrow-derived Kupffer cells; CLs, clodronate liposomes; E, embryonic days; DTR, diphtheria toxin receptor; EMP, erythro-myeloid progenitors; Em-KCs, embryo-derived Kupffer cells; HSC, hepatic stellate cell; HBV, hepatitis B virus; HCC, hepatocellular carcinoma; KCs, Kupffer cells; LDTF, lineage determining transcription factor; LPS, lipopolysaccharide; LIRI, liver ischemia-reperfusion injury; LSEC, liver sinusidal endothelial cells; NASH, non-alcoholic steatohepatitis; scRNA-seq, Single cell RNA-sequencing; YS, yolk sac.
